# Provision of healthcare services for displaced individuals globally is a pressing concern

**DOI:** 10.3389/fpubh.2024.1344984

**Published:** 2024-01-29

**Authors:** Ahmed Hossain

**Affiliations:** College of Health Sciences, University of Sharjah, Sharjah, United Arab Emirates

**Keywords:** displacement, refugee, migration, health, healthcare

The United Nations Refugee Agency (UNHCR) paints a stark picture of forced displacement: a staggering 108.4 million people were uprooted by the end of 2022, a record high representing a surge of 19.1 million from the previous year (1). This includes 35.3 million refugees, 53.2 million internally displaced, and 4.9 million asylum seekers (1). This staggering number eclipses even the mass displacement of World War II (1945), marking a tragic new chapter in global displacement. The current article shed light on the worldwide healthcare circumstances faced by displaced populations, offering significant perspectives on the hidden complexities associated with this matter.

The world faces a displacement crisis of staggering proportions, driven by armed conflict, physical aggression, persecution, and natural disasters. Recent data reveals the five main countries generating refugees: Syria, Venezuela, Afghanistan, South Sudan, and Myanmar (1). Meanwhile, Turkey, Colombia, Pakistan, Uganda, and Iran host the most (1). But the causes go beyond human conflict. Environmental factors, particularly devastating natural disasters and trigger internal displacement. In 2022, Pakistan endured the brunt of this, with 8.2 million people internally displaced due to disasters (2). This surging tide of displacement presents a critical crossroads for healthcare systems and practitioners. Bridging the gap between diverse needs, heightened vulnerabilities, and strained resources demands innovative solutions and unwavering commitment.

In Cox's Bazar, Bangladesh, housing one of the largest refugee camps globally, the Rohingya community heavily relies on humanitarian aid, especially the food rations distributed by the World Food Programme (WFP). The reduction in these rations for the nearly one million Rohingya residents drew criticism from the United Nations in Bangladesh on June 1, 2023 (3). The decrease in assistance to these refugees may be attributed to factors such as the COVID-19 pandemic, the conflict in Ukraine, and a significant rise in food costs. The World Food Programme urgently appeals for funding to support the global refugee community, where severe hunger is already prevalent (4). Similar situations are observed among Syrian refugees in Turkey, Lebanon, and Jordan (5) and among refugees from Burundi and Congo in Tanzania (6). Providing healthcare services to refugees and host populations in host countries necessitates the international community's acknowledgment of shared responsibility, which should include both financial commitment and intention (3, 6).

The COVID-19 pandemic has cast a long shadow over the lives of refugees, compounding their existing vulnerabilities and creating a triple burden on their physical, mental, and social wellbeing (7, 8). While the COVID-19 pandemic has undoubtedly exacerbated the challenges faced by refugees, it has also highlighted the need for stronger international cooperation and a renewed commitment to upholding the rights of displaced people. By addressing the triple burden on their health and wellbeing, we can build a more inclusive and resilient future for refugees worldwide.

Displacement can have a devastating impact on individuals' wellbeing, with the consequences reaching far beyond just physical health. Refugees often face numerous challenges, including economic hardship, food insecurity, restricted access to healthcare, and instances of discrimination (4–7). Past traumatic experiences may further hinder their ability to adapt to their current circumstances (9). Beyond the myriad challenges of adjusting to a new country, individuals may have endured conflict, violence, multiple losses, torture, sexual abuse, and the potential for exploitative situations. They may be vulnerable to various forms of gender-based violence, domestic violence (within families or spousal relationships), honor-based violence, trafficking or forced displacement, modern slavery, and forced marriages. In addition to immediate political and economic obstacles, addressing this humanitarian crisis necessitates health-related responses, particularly in the realm of mental health policies and interventions.

Refugees and migrant populations need comprehensive support to improve their overall health. This support should encompass access to nutrition, hydration, housing, sanitation, healthcare, education, and vocational training. It is crucial to consider patients' preferences regarding the language, gender, and cultural background of healthcare providers, as these factors can significantly impact trust and information disclosure. Making every effort to establish clear and effective patient communication is essential. For pregnant individuals, ensure access to antenatal services, provide maternal vitamins (such as folic acid, vitamins C, and D), promote breastfeeding, offer contraception, and discuss the importance of cervical cancer screening.

Many nations globally are presently grappling with a notable humanitarian crisis, necessitating aid to fulfill the exacting requirements of their complex healthcare responsibilities. Primarily, it is imperative that individuals are provided with required nutrition and medical attention in the present moment. Nonetheless, it is equally essential to contemplate their future possibilities.

## Author contributions

AH: Conceptualization, Investigation, Resources, Validation, Visualization, Writing—original draft, Writing—review & editing.
